# Discovery of Two Brominated Oxindole Alkaloids as Staphylococcal DNA Gyrase and Pyruvate Kinase Inhibitors via Inverse Virtual Screening

**DOI:** 10.3390/microorganisms8020293

**Published:** 2020-02-20

**Authors:** Ahmed M. Sayed, Hani A. Alhadrami, Seham S. El-Hawary, Rabab Mohammed, Hossam M. Hassan, Mostafa E. Rateb, Usama Ramadan Abdelmohsen, Walid Bakeer

**Affiliations:** 1 Department of Pharmacognosy, Faculty of Pharmacy, Nahda University, Beni-Suef 62513, Egypt; ahmed.mohamed.sayed@nub.edu.eg; 2 Department of Medical Laboratory Technology, Faculty of Applied Medical Sciences, King Abdulaziz University, Jeddah 21589, Saudi Arabia; hanialhadrami@kau.edu.sa; 3 King Fahd Medical Research Centre, King Abdulaziz University, Jeddah 21589, Saudi Arabia; 4 Department of Pharmacognosy, Faculty of Pharmacy, Cairo University, Cairo 11787, Egypt; seham.elhawary@yahoo.com; 5 Department of Pharmacognosy, Faculty of Pharmacy, Beni-Suef University, Beni-Suef 62514, Egypt; rmwork06@yahoo.com (R.M.); abuh20050@yahoo.com (H.M.H.); Mostafa.Rateb@uws.ac.uk (M.E.R.); 6 School of Computing, Engineering & Physical Sciences, University of the West of Scotland, Paisley PA1 2BE, UK; 7 Department of Pharmacognosy, Faculty of Pharmacy, Minia University, Minia 61519, Egypt; usama.ramadan@mu.edu.eg; 8 Department of Pharmacognosy, Faculty of Pharmacy, Deraya University, Minia 61111, Egypt; 9 Department of Microbiology, Faculty of Pharmacy, Beni-Suef University, Beni-Suef 62514, Egypt

**Keywords:** MRSA, trisindoline, antibacterial, antibiofilm, inverse virtual screening, gyrase-B, pyruvate kinase, marine natural products

## Abstract

In the present study, a small marine-derived natural products library was assessed for antibacterial potential. Among 36 isolated compounds, a number of *bis*-indole derivatives exhibited growth-inhibitory activity towards Gram-positive strains (*Bacillus subtilis* and multidrug-resistant *Staphylococcus aureus*). 5- and 6-trisindoline (5-Tris and 6-Tris) were the most active derivatives (minimum inhibitory concentration, MIC, 4–8 µM) that were subsequently selected for anti-biofilm activity evaluation. Only 5-Tris was able to inhibit the staphylococcal biofilm formation starting at a 5 µM concentration. In order to investigate their possible molecular targets, both natural products were subjected to in silico inverse virtual screening. Among 20 target proteins, DNA gyrase and pyruvate kinase were the most likely to be involved in the observed antibacterial and anti-biofilm activities of both selected natural products. The in vitro validation and in silico binding mode studies revealed that 5-Tris could act as a dual enzyme inhibitor (IC_50_ 11.4 ± 0.03 and 6.6 ± 0.05 µM, respectively), while 6-Tris was a low micromolar gyrase-B inhibitor (IC_50_ 2.1 ± 0.08 µM), indicating that the bromine position plays a crucial role in the determination of the antibacterial lead compound inhibitory activity.

## 1. Introduction

**Figure 1:**
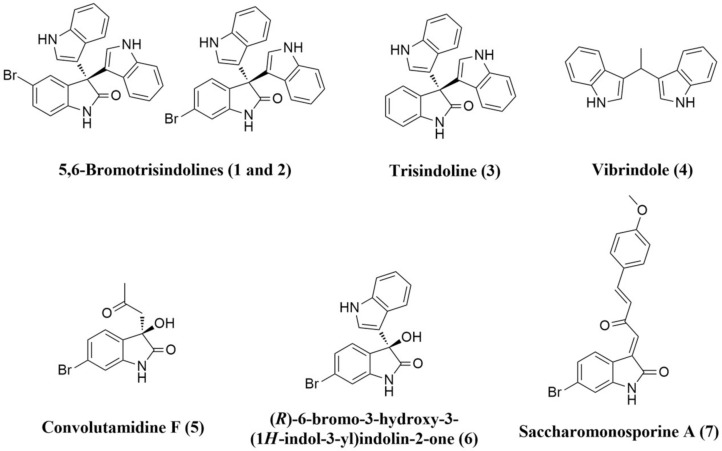
Some examples of marine-derived indole compounds.

**Figure 2:**
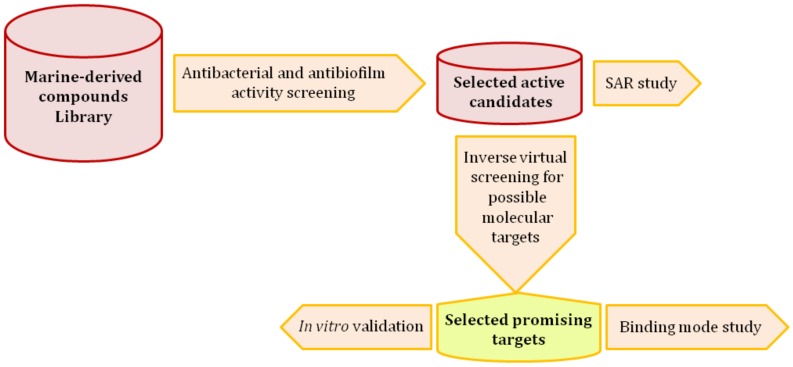
Outline of the procedure used in this study. SAR: structure–activity relationship.

Staphylococci are considered dangerous opportunistic microorganisms, and are associated with many serious infections, including those that are hospital-acquired. In most cases of staphylococcal nosocomial infections, the isolated causative strains were found to be antibiotic-resistant [1,2,3]. The number of reported multidrug-resistant *Staphylococcus aureus* and *Staphylococcus epidermidis* (MRSA and MRSE, respectively) strains that show resistance against conventional antibiotics (e.g., macrolides and aminoglycosides) is on the rise. Additionally, their ability to protect themselves within biofilms on many different surfaces significantly contributes to antibiotic-resistance (i.e., phenotypic resistance) [4]. The limited effectiveness of standard antibiotics in dealing with biofilm-related infections, which increase the emergence of multidrug-resistant staphylococci, together with the increased dependence on implanted medical devices, drives the need for exploring and developing new classes of antimicrobials. Recently, pyruvate kinase (PK) has been identified as a crucial enzyme in staphylococci, and regulates their growth, antibiotic resistance, and biofilm formation [5,6,7]. Furthermore, it was found to be structurally distinct from human homologs, and hence it provides a promising target for novel antimicrobial agents [8,9]. On the other hand, DNA gyrase is a topoisomerase-type enzyme that is required during bacterial DNA replication and transcription to maintain topology and integrity. It consists of four subunits (two A subunits and two B subunits) attached together to form a tetrameric holoenzyme [10]. Currently, DNA gyrase is considered one of the primary targets and has been clinically validated in most pathogenic bacteria. Fluoroquinolones are well-known DNA gyrase inhibitors that target the enzyme’s A subunit specifically. They have achieved great success as antibacterial agents over the last 20 years; however, a number of resistant strains have emerged recently. Additionally, they are more active towards the Gram-negative bacteria in comparison with Gram-positive ones such as *S. aureus* and *S. epidermidis* [11]. Many research groups worldwide are focusing on the development of novel antibacterial drugs by targeting DNA gyrase B subunits [12,13,14]. Besides their bactericidal activity, DNA gyrase inhibitors have recently been identified as active anti-biofilm agents toward a wide range of pathogenic bacteria, most notably staphylococci (MRSA and MRSE) [15]. Natural products derived from marine sources have been showing promising pharmacological effects towards a wide array of molecular targets, and their unusual structural diversity provides a crucial source of lead compounds [16]. We previously reported a number of bioactive metabolites from the Red Sea-derived sponge *Callyspongia siphonella* and its associated microorganisms (Table S1) [17,18,19]. Notably, the two alkaloids, 5- and 6-bromotrisindoline (5-Tris and 6-Tris, respectively; Compounds 1 and 2, Figure 1) together with some phenolic acid derivatives (Compounds 16–27, Table S1) have shown in vitro antibacterial and antitrypanosomal activities, and cytotoxicity against several tumor cell lines. Moreover, the alkaloids saccharomonosporine A and convolutamydine F (Compounds 8 and 11) were identified as potent cytotoxic Pim-1 kinase inhibitors. In the present study, as part of our interest in the discovery of new drug leads from marine-derived natural products, we screened a small marine natural products library (Compounds 1–36, Table S1) to determine their potential as antibacterial and antibiofilm agents against staphylococci. Subsequently, the most active compounds were subjected to in silico inverse virtual screening and in vitro assays to detect their possible molecular targets. The working outline is depicted in Figure 2.

## 2. Material and Methods

### 2.1. Library Construction

All compounds used in the present study (Compounds 1–36, Table S1, Supplementary Materials) were isolated from the Red Sea-derived sponge *Callyspongia siphonella* and its associated microorganisms using multiple chromatographic purification steps and using preparative HPLC (Agilent^®^ 1260 Infinity, CA, USA) as a final step to obtain these compounds in pure form. The detailed procedure of compounds extraction and isolation were described previously [17,18,19]. All chemical structures of the isolated natural metabolites were determined by analysis of their spectral data (MS and NMR) and comparison with reported literature. The Bruker Avance III 400 MHz (Bruker AG, Switzerland) and Agilent series 1100 SL (Agilent CO, Santa Clara, CA, USA) were used to acquire the NMR and MS spectra, respectively. The produced library consisted of 15 indole derivatives, 12 phenolic derivatives, 6 steroidal compounds, 2 triterpenoid compounds, and 1 fatty acid.

### 2.2. Bacterial Strains

Four human pathogenic bacteria were used in this study, MRSA ATCC33591, *Staphylococcus epidermidis* RP62A ATCC12228, *Escherichia coli* ATCC 259228, and *Pseudomonas aeruginosa* ATCC9027, in addition to the non-pathogenic Gram-positive *Bacillus subtilis* ATCC5230. All the bacterial strains were maintained at −80 °C in 15% glycerol nutrient broth (Oxoid, Milan, Italy).

### 2.3. Determination of Minimum Inhibitory Concentration (MIC)

The twofold serial dilution approach was applied to determine the MICs of each compound. Each tested compound was dissolved in DMSO (Sigma, Milan, Italy) to prepare the test solutions. Many colonies of each tested bacterial strain were inoculated in 10 mL of sterilized Mueller–Hinton broth (MHB) (Oxoid) and incubated at 37 °C for 18–24 h. Subsequently, each bacterial suspension was adjusted to a final concentration of 100 cfu/mL (OD_595_ 0.13–0.15); then 100 µL of the bacterial suspension were added in the 96-well cell culture plate (Cellstar^®^, Greiner Bio-One, Frickenhausen, Germany), with 0.5 to 128 µM of the test compound solutions. The optical density (595 nm) of every well was measured using a Multiscan Ex Microplate Reader (Thermo Scientific, Germany). MIC values were estimated as the lowermost compound concentration that inhibits the bacterial growth after 24 h of incubation. All data are reported as the mean of three independent experiments.

### 2.4. Biofilm Assay

The biofilm assay was performed in a 96-well polystyrene plate with a flat bottom according to the previously described method [2]. *S. epidermidis* RP62A (OD_595_ ~ 0.05 in TSB) was incubated in with 5-Tris and 6-Tris at different concentrations at 37 °C for 24 h. OD_595_ readings were used to estimate the MICs. Then, the test compound concentration where the lowest OD_595_ values were measured with no visible bacterial growth was used to calculate the MIC. After OD_595_ measurements, the bacterial cells were discarded by rinsing with sterile PBS. Then, the biofilm cells were fixed by heat at 65 °C for 1 h. Plates were subsequently stained with 0.3% crystal violet for 5 min, and then rinsed three times with sterile double-distilled water and air dried. Finally, OD_492_ values were used to determine the magnitude of biofilm inhibition in comparison with the control in test wells. *S. epidermidis* (ATCC12228) was used in the experiment as the biofilm negative strain.

### 2.5. In Vitro Enzymes Assay

DNA gyrase (type II topoisomerase) and topoisomerase IV (Topo IV; ParE) introduce negative supercoils into DNA, utilizing ATP hydrolysis as a source of energy. They are considered essential bacterial regulatory enzymes that are absent in eukaryotes. Gyrase subunit B (Gyr-B) and Topo IV inhibitory activities were determined using the Inspiralis assay kit (Inspiralis^®^, UK) on streptavidin-coated 96-well microtiter plates (Thermo Scientific, Germany) according to the manufacturer’s protocols [20]. The assay measures the ability of the tested compounds to inhibit the ATPase activity of both Gyr-B and ParE subunits. Briefly, the plates were hydrated with buffer (20 mM Tris-HCl with pH 7.6, 0.01% *w*/*v* BSA, 0.05% *v*/*v* Tween 20, 137 mM NaCl) and the biotinylated oligonucleotide was then immobilized. After the unbound oligonucleotide was washed out, the enzyme inhibitory assay was performed. The reaction volume of 30 μL in buffer (35 mM Tris × HCl with pH 7.5, 4 mM MgCl_2_, 24 mM KCl, 2 mM DTT, 1.8 mM spermidine, 1 mM ATP, 6.5 % *w*/*v* glycerol, 0.1 mg/mL albumin) contained 1.5 U of DNA gyrase or Topo IV from *S. aureus*, 0.75 μg of relaxed pNO1 plasmid, and 3 μL solution of the inhibitor in 10% DMSO and 0.008% Tween 20. Subsequently, the reaction solutions were incubated at 37 °C for 30 min. At the end of the reaction, the TF buffer (50 mM NaOAc with pH 5.0, 50 mM NaCl, and 50 mM MgCl_2_) was added. After additional incubation for 30 min at room temperature, during which the biotinoligonucleotide-plasmid triplex was formed, the unbound plasmid was washed off using TF buffer (10 mM Tris HCl with pH 8.0 and 1 mM EDTA). The produced fluorescence was measured using a microplate reader (BioTek Synergy, excitation: 485 nm, emission: 535 nm, Germany). Initial screening was done at 100 or 10 μM concentration of inhibitors. PK has recently been discovered as an essential hub-protein in the interactome of MRSA [21]. The PK inhibitory activity of the tested compounds was determined according to the previously reported method [22]. Shortly, MRSA PK was expressed in pET-28a (+) as a recombinant protein in *E. coli* BL-21(DE3) and then purified by using Ni-nitrilotriacetic acid (NTA) agarose (Quiagen, Inc., Germantown, MD). The PK activity was measured in a continuous assay coupled to lactate dehydrogenase (LDH) in which the change in absorbance at 340 nm owing to the oxidation of NADH was measured by the microplate reader. The reaction mixture contained 60 mM Na^+^-HEPES (pH 7.5), 5% glycerol, 67 mM KCl, 6.7 mM MgCl2, 0.24 mM NADH, 5.5 units LDH from rabbit muscle (Sigma Aldrich), 2 mM ADP, and 10 mM PEP in a total volume of 200 mL. The reaction was initiated by addition of the PK (15 nM). Inhibitors were dissolved in dimethyl sulfoxide (DMSO) with the final concentration of the solvent never exceeding 1% of the whole assay volume. IC_50_ values in both assays were determined using seven concentrations of tested compounds. GraphPad Prism software was used to calculate the IC_50_ values. The result was given as the average value of three independent measurements.

The inhibition constant *Ki* values for 5-Tris and 6-Tris were determined according to the manufacturer’s protocols, where the rate of chromogenic substrate hydrolysis, using 2 mM of the tested enzyme and 0–250 µM of the substrate, was monitored in increasing amounts of inhibitor (0–10 nM).

### 2.6. In Silico Inverse Screening

Well-characterized protein targets, known to be essential for *Staphylococcus* growth and biofilm formation, were selected in the Protein Data Bank (PDB) database. Subsequently, these targets were prepared by removing the water molecules and with the addition of polar hydrogens with Autodock Tools 1.4.5. Autodock Vina software was used for docking calculations. The grid volumes used for binding calculations were built using the co-crystallized ligands as a reference (Table S2, Supplementary Materials). An exhaustiveness of 16 was used for the docking studies. Autodock Vina results were analyzed with Pymol software.

### 2.7. Docking Analysis

Staphylococcal Gyr-B and PK crystal structures of the of PDB codes 3g7b and 3T0T, respectively, were used. Docking experiments were performed using AutoDock Vina docking software. Such docking engines deal with the receptor as a rigid structure and the ligand as a flexible structure during its calculations. The co-crystallized ligands were utilized to assign the binding sites. The ligand-to-binding-site shape matching root mean square threshold was set to 2.0 Å. The interaction energies were determined using the CFF force field (v.1.02) with 10.0 Å as a non-bonded cutoff distance and distance-dependent dielectric. Then, 5.0 Å was set as an energy grid extending from the binding site. The tested compounds were energy-minimized inside the selected binding pocket. The editing and visualization of the generated binding poses were performed using Pymol software.

### 2.8. ADME Studies

ADME profiling was measured in silico using the online website “http://www.swissadme.ch/”. Gastrointestinal (GIT) absorption, blood–brain barrier (BBB), solubility, bioavailability score, and inhibition of CYP2D6 were selected as ADME descriptors to be calculated.

### 2.9. Toxicity Profiling

Toxicity profiling was estimated using PreADMET software version 2.0. We screened the active compounds for carcinogenicity (rat and mouse), mutagenicity, and in vitro hERG inhibition (cardiotoxicity).

### 2.10. Statistical Analysis

All the experiments were performed in triplicates. Data were expressed as mean ± SEM. *p*-Values < 0.05 were considered as statistically significant. GraphPad Prism^®^ version 6.01 was used for the statistical analysis of experimental data.

## 3. Results and Discussion

### 3.1. Inhibitory Activity Screening

The antibacterial evaluation was performed for our small natural products library (Compounds 1–36, Table S1) in terms of MIC values in µM using standard antibacterial agents (ampicillin and gentamicin). The results of inhibitory evaluation along with our previous inhibitory reports on other bacterial strains [19] illustrated that a number of related indole derivatives were the only active compounds. The brominated oxindole derivatives, 5-bromotrisindoline and 6-bromotrisindoline (5-Tris and 6-Tris, respectively; Compounds 1 and 2; Table S1) were the most potent metabolites against Gram-positive bacterial MRSA (MIC 8 and 4 µM, respectively) and *B. subtilis* (MIC 4 and 4 µM/mL, respectively). The remaining compounds in this small compound library (phenolics, sterols, and triterpenes) were inactive against all tested organisms.

### 3.2. Antibiofilm Activity

**Figure 3:**
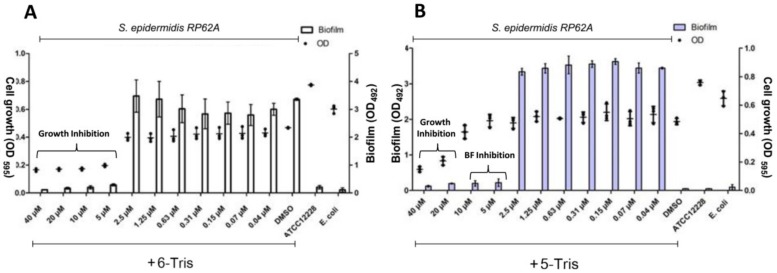
Inhibition of biofilm formation and planktonic growth of *Staphylococcus epidermidis* RP62A by (**A**) 5-trisindoline (5-Tris) and (**B**) 6-trisindoline (6-Tris). OD: Optical Density.

To further evaluate the anti-biofilm activity of 5-Tris and 6-Tris in Gram-positive bacteria, they were tested at different concentrations against the reference strain *S. epidermidis* RP62A [2,22]. As shown in Figure 3, both molecules reduced the growth of an *S. epidermidis* standard strain in a dose-dependent manner. We found that 6-Tris has strong antibacterial activity only, where it inhibited the bacterial growth up to a 5-µM concentration. At lower concentrations, it did not show any effect on both growth and biofilm formation. On the other hand, 5-Tris revealed lower growth inhibitory activity, where it inhibited the bacterial growth starting at 20 µM. Moreover, it completely prevented both standard strains from forming their biofilm at sub-MIC concentrations until 5 µM. We can conclude from these results that 6-Tris is more potent than its isomer 5-Tris in terms of growth inhibitory activity (MIC 5 µM and 20 µM, respectively). However, 5-Tris demonstrated a significant antibiofilm activity with minimum biofilm inhibitory concentration (MBIC) of 5 µM (0.25 MIC). Furthermore, the results suggested that the position of the bromine atom in 6-position favored the antibacterial activity; however, the 5- position favored the antibiofilm properties.

### 3.3. Structure–Activity Relationship (SAR) Study

**Figure 4:**
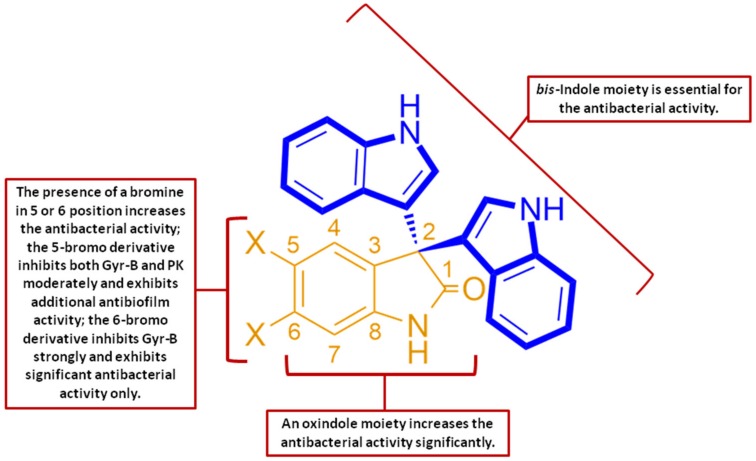
Structure–activity relationship of the bioactive indole compounds. PK: pyruvate kinase; Gyr-B: gyrase subunit B.

From both the antibacterial (Table S1) and antibiofilm results (Figure 3), we can conclude that the *bis*-indole is an essential moiety for the antibacterial activity against Gram-positive bacteria. The addition of an extra oxindole group to form a tris-indole scaffold doubled the antibacterial activity. Moreover, the presence of a bromine atom significantly increased the activity (4–8 fold). The 5-bromo derivative 5-Tris was the only active compound as a biofilm inhibitor, indicating that the position of the bromine in carbon 5 is essential for the antibiofilm activity (Figure 4). Both oxindoles (5-Tris and 6-Tris) may exhibit a narrow spectrum of antibacterial activity (Gram-positive only) as a result of being neutral compounds. Such molecules together with negatively charged ones were shown to be unable to cross the protective outer membrane of Gram-negative bacteria [23]. Several previous studies have illustrated that the positively charged antibiotics, particularly those containing primary amine groups, accumulate preferentially in Gram-negative bacteria, and hence, during the future development of these lead compounds, their activity spectrum could be expanded through the addition of a suitable primary amine group [24].

### 3.4. Inverse Virtual Screening

Molecular docking studies were performed for both 5-Tris and 6-Tris to identify potential protein targets [25]. A number of molecular targets that are common in staphylococci [26,27] were downloaded from the protein database (PDB) and prepared for the docking experiment. Table 1 arranges the binding energies in kcal/mol of the two compounds against a list of possible targets. 5-Tris and 6-Tris showed the highest predicted affinity toward both DNA gyrase subunit B (Gyr B) and pyruvate kinase (PK) (binding energy of −7.3 and −9.9 kcal/mol for Gyr B, and −8.8 and −6.9 kcal/mol for PK, respectively) among the docked molecular targets, and hence they were selected for the subsequent in vitro evaluation.

### 3.5. In Vitro Assay

From the in silico screening results, we hypothesized that both Gyr B and PK could be potential targets for 5-Tris and 6-Tris. Inhibition of DNA gyrase subunit A (Gyr A) leads to direct cell death by trapping the gyrase–DNA complex. On the other hand, inhibition of Gyr B deprives the energy source needed for DNA replication [14]. Gyr B as a molecular target offers a chance to avoid the cross-resistance to the well-known Gyr A inhibitors, quinolones. PK is known to be a crucial enzyme that catalyzes the final glycolysis step, which includes a single phosphoryl group transfer from phosphoenolpyruvate to ADP, to produce a molecule of pyruvate and ATP [28]. Recently, it was considered as a “superhub” protein in *S. aureus* and was found to be essential for staphylococcal growth and biofilm formation [5,6,7]. In vitro studies (Table 2) showed 6-Tris to be a potent Gyr B inhibitor (IC_50_ 2.1 ± 0.08 µM) with weak inhibitory activity against PK (IC_50_ 23.2 ± 0.06 µM). On the other side, 5-Tris exhibited significant inhibitory activity against PK (IC_50_ 6.6 ± 0.05 µM) and lower activity towards Gyr B (IC_50_ 11.4 ± 0.03 µM) than 6-Tris. Regarding the enzyme affinity of both compounds (Table 2), 5-Tris showed much more affinity towards PK than 6-Tris (*Ki*: 5.6, 21.9 µM, respectively), but less affinity towards Gyr-B than 6-Tris (*Ki*: 7.75, 1.5 µM, respectively). These results clearly illustrated the role of the bromine position in the selectivity and potency of these inhibitors. Furthermore, they can suggest that the PK inhibition may have a direct link to the antibiofilm activity that was exhibited by 5-Tris. Earlier reports supported this point of view and explained the crucial role of PK in the staphylococcal biofilm formation [6]. To further validate the in silico prediction accuracy we tested 5-Tris and 6-Tris against staphylococcal topoisomerase IV (ParE, Topo IV), which has an essential role in DNA replication similar to DNA gyrase (Gyr-B), but showed significantly lower predicted binding affinity towards both 5-Tris and 6-Tris (Table 1). The assay results supported the predicted ones and revealed that both compounds have weak inhibitory activity against Topo IV, where 5-Tris and 6-Tris were 3 times and 12 times more active against Gyr-B than Topo IV, respectively (IC_50_ 33.17 ± 0.04 and 25.14 ± 0.03 µM, respectively).

### 3.6. In Silico Binding Mode Study

**Figure 5:**
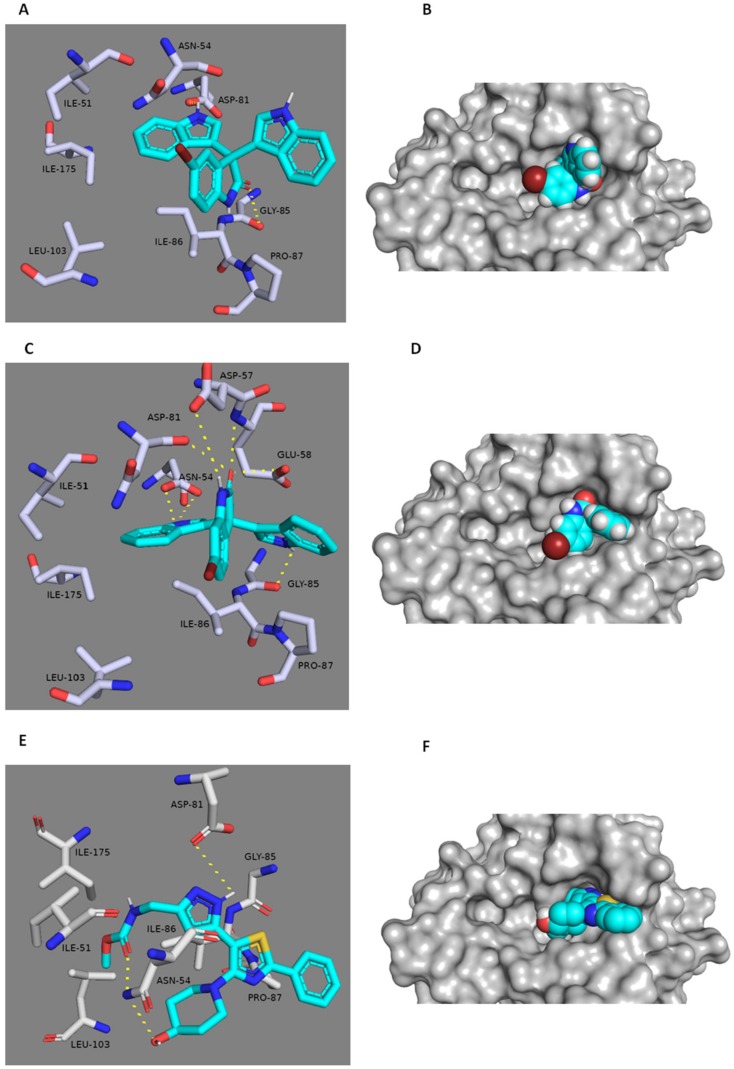
Docking study of 5-Tris (**A**,**B**) and 6-Tris (**C**,**D**) within the active site of multidrug-resistant *Staphylococcus aureus* (MRSA) Gyr-B. The key binding interactions of the Gyr-B co-crystallized ligand [29] are shown in (**E**,**F**). The amino acid side chains are depicted in (**A**,**C**,**E**) for clarification.

**Figure 6:**
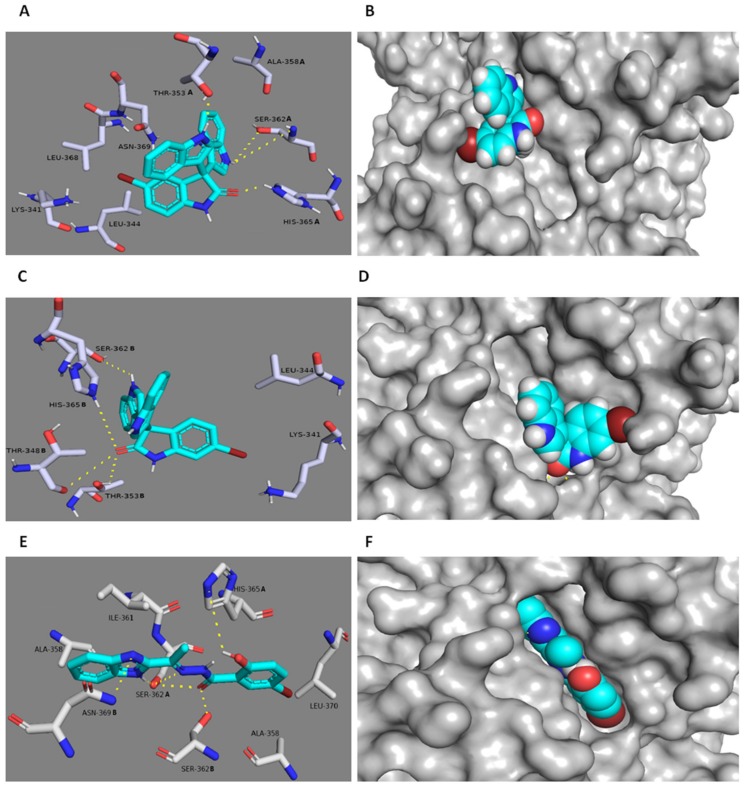
Docking study of 5-Tris (**A**,**B**) and 6-Tris (**C**,**D**) within the active site of MRSA PK. The key binding interactions of the PK co-crystallized ligand [21] are shown in (**E**,**F**). The amino acid side chains are depicted in (**A**,**C**,**E**) for clarification.

The potential binding modes of 5-Tris and 6-Tris with both Gyr B and PK were analyzed by docking their active binding sites. MRSA Gyr B and PK of PDB code (3g7b and 3T0T) were chosen for the docking experiments due to their optimum resolutions (2.3 Å and 3.1 Å) and are co-crystallized with their inhibitors. The spheres around the co-crystallized inhibitors were assigned as binding sites for docking. Both 5-Tris and 6-Tris showed convergent docking poses (Figure 5) and were comparable to the co-crystallized Gyr-B inhibitor [29]. The indole nitrogen in only one indole group of the two 5-Tris indoles is hydrogen-bonded to the carbonyl oxygen of ASP-81, and the benzene moiety of the same indole is buried within a hydrophobic pocket of ILE-51, ILE-175, and LEU-103. Moreover, the carbonyl oxygen of the oxindole is hydrogen-bonded to the main chain carbonyl of GLY-85. The co-crystallized ligand forms a similar hydrogen bond with ASP-81 and a further two hydrogen bonds with a different amino acid residue, ASN-54. Alternatively, the existence of bromine in position 6 in 6-Tris directed the whole molecule toward a better binding mode and better fitting inside the binding pocket of Gyr-B (Figure 5). Both indole groups of the molecule became able to interact with the binding site’s residues, where the nitrogen of the first one is similarly hydrogen-bonded to the carbonyl and hydroxyl oxygen of ASP-81, and the nitrogen of the second indole is hydrogen-bonded to GLY-85. Furthermore, both the amidic hydrogen and the carbonyl oxygen of the oxindole moiety are tightly anchored inside the binding cavity through four hydrogen bonds with ASP-57 and GLU-58, respectively, and through the reported one with ASN-54. Such a strong interaction can explain the superior activity of 6-Tris (IC_50_ 2.1 ± 0.08 µM) over 5-Tris (IC_50_ 11.4 ± 0.03 µM) and the co-crystallized ligand. On the other hand, both 5-Tris and 6-Tris showed different binding modes in the PK active site. The co-crystalized PK inhibitor [21,30] is perfectly fitted inside the pocket formed between the enzyme’s subunits A and B (Figure 6). This binding mode is stabilized by six main hydrogen bonds with SER-362A, SER-362B, ASN-369B, and HIS-365A (A and B correspond to PK subunits). These residues provide target selectivity because they are conserved only in the staphylococcal PK and not found in the human PK. Herein, 5-Tris shows convergent binding mode to the co-crystallized PK inhibitor, where it anchors in one side of the PK active site (Figure 6) through three hydrogen bonds with SER-362A and HIS-365A, similarly to the co-crystalized ligand, and an additional hydrogen bond with THR-353A. Moreover, the 5-bromo group is further stabilized 5-Tris binding through the strong hydrophobic interaction with the hinge of LEU-368B, LYS-341B and LEU-344B (Figure 6). Regarding 6-Tris, again, the position of the bromine atom shows a vital role in the interaction with the active site. The bromine in 6-Tris directed the whole molecule to interact with the other side of the binding pocket (Figure 6), where the carbonyl oxygen of the oxindole moiety forms three weak hydrogen bonds (distance > 4 Å) with HIS-365B, THR-348B, and THR-353B, in addition to another weak hydrogen bond between the nitrogen of one indole moiety with SER-362B, and thus this weak interaction in comparison to 5-Tris explains the inferior activity against PK (5-Tris IC_50_ = 6.6 ± 0.05 µM; 6-Tris IC_50_ = 23.2 ± 0.06 µM).

### 3.7. In Silico ADME/Tox Prediction

The majority of drug candidates fail during the clinical development due to inappropriate ADME/Tox profiles, and hence, the virtual screening should not be limited to predicting and optimizing binding affinity of a given drug molecule, but the pharmacokinetic parameters should also be involved as significant filters to further optimize selected lead molecules into drug candidates, and reduce failure rates during clinical trials. The predicted ADMET/Tox profiles of 5-Tris and 6-Tris were calculated using the online software SwissADME and preADMET. Generally, both compounds showed excellent drug-like properties, high oral absorption, high bioavailability, and moderate toxicity (Table 3 and Table 4). Such predicted toxicity (e.g., the mutagenic and carcinogenic characteristics) of both compounds should be taken into consideration during their development, so that the possible future derivatives will be more efficient as therapeutic agents with minimal toxicity profiles.

## 4. Conclusions

Currently, much research is being directed toward the discovery of novel antimicrobial molecules that can act as biofilm inhibitors. Marine invertebrates are considered a prolific source of diverse pharmacologically active metabolites with different ecological potentials. In the present investigation, we prepared a small library of bioactive metabolites that were isolated from the marine-derived sponge *C. siphonella* and its associated symbiotic microbes. This library was screened against a panel of standard bacterial pathogens (two Gram-positive and two Gram-negative strains), including MRSA. Based on the obtained results, both 5-Tris and 6-Tris were the most active compounds and subsequently were selected for antibiofilm activity evaluation against a standard strain (*S. epidermidis*). Only 5-Tris was able to inhibit the ability of *S. epidermidis* to form its biofilm up to 5 µM. On the other hand, 6-Tris showed strong bacterial growth activity without biofilm inhibition. The antibacterial and antibiofilm results enabled us to construct a structure–activity relationship for the *bis*-indole scaffold. To investigate the possible molecular targets that may mediate these biological effects, we screened both 5-Tris and 6-Tris against a panel of selected targets (inverse virtual screening). This innovative in silico approach allowed us to prioritize Gyr-B and PK as the most promising candidates depending on the binding energy of each target protein. The in vitro assay thereafter revealed that 6-Tris is a potent Gyr-B inhibitor with weak activity against PK; however, 5-Tris is considered a dual inhibitor of both enzymes with a moderate potency. Additionally, the in silico ADME predictions suggested that both 5-Tris and 6-Tris have drug-like properties and could be promising leads for further evaluation. Further development and in vivo studies will be of interest to improve the potency and to assess the efficacy as well as the possible toxicity of these molecules.

## Figures and Tables

**Table 1:** Binding energies (kcal/mol) of 5-Tris and 6-Tris against a number of possible staphylococcal targets.

Molecular Target	Function	Mode of Action	Binding Energy (5-Tris)	Binding Energy (6-Tris)
FtsZ	GTPase	Cell division	−6.3	−6.0
PK	Pyruvate kinase	Glycolysis	−8.8	−6.9
SpsB	Signal Peptidase	Protein secretion	−3.9	−5.0
Isoleucyl-tRNA synthetase	Protein biosynthesis	Protein modification	−5.3	−4.0
Pdf	Peptide deformylase	Protein modification	−5.8	−4.9
Peptidyl transferase	Protein biosynthesis	Protein modification	3.9	4.1
rRNA methyltransferase	Protein biosynthesis	Protein modification	−4.2	−5.5
Threonyl−tRNA synthetase	Protein biosynthesis	Protein modification	−5.1	−5.2
Gyr A	DNA gyrase	DNA replication	−4.3	−4.8
Gyr B	DNA gyrase	DNA replication	−7.3	−9.9
ParE	Topoisomerase IV	DNA replication	−5.4	−5.1
Ddl	D-alanine ligase	Peptidoglycan synthesis	−4.1	−4.8
MurB	UDP-N-acetylglucosamine-enolpyruvyl reductase	Peptidoglycan synthesis	−6.1	−5.5
PBP2	Peptidoglycan glycosyl transferase	Peptidoglycan synthesis	−3.6	−3.8
DHFR	Dihydrofolate reductase	Cellular regulation	−6.9	−6.1
YycG/YycF	Autolysis	Cellular regulation	−6.7	−6.2
FabF	B-ketoacyl-synthase I/II	Fatty acid synthesis	−3.6	−3.5
FabI	Enoly-acyl-carrier protein reductase	Fatty acid synthesis	−4.3	−4.2
LigA	DNA ligase	Stress response	−7.0	−6.1
TrxB	Thioredoxin reductase	Stress response	−5.9	−5.0

**Table 2:** MRSA DNA gyrase-B, pyruvate kinase, and topoisomerase IV inhibitions as IC_50_ and inhibition constant *Ki* values.

Tested Compound	IC_50_ (*Ki*) ± S.D. µM ^a^		
	DNA Gyrase	Pyruvate Kinase	Topoisomerase IV
**5-Tris**	11.4 ± 0.03 (*Ki*: 7.75 ± 0.05)	6.6 ± 0.05 (*Ki*: 5.6 ± 0.02)	33.17 ± 0.04
**6-Tris**	2.1 ± 0.08 (*Ki*: 1. 5 ± 0.03)	23.2 ± 0.06 (*Ki*: 21.9 ± 0.04)	25.14 ± 0.03
**Novobiocin**	0.12 ± 0.01	*	0.22 ± 0.01

^a^ Values are the mean of three independent experiments. *—not determined.

**Table 3:** Predicted ADME profiles of 5-Tris and 6-Tris.

Compound	Lipinski ^a^	BBB ^b^	GIT Absorption ^c^	Solubility ^d^	CYP2D6 ^e^	Bioavailability Score ^f^
**5-Tris**	Yes	Yes	High	Moderate	Yes	0.55
**6-Tris**	Yes	Yes	High	Moderate	Yes	0.55

^a^ Predicts if the compound has a drug-like properties (follows the Lipinski rule of five); ^b^ Predicts the ability of the compound to penetrate the blood–brain barrier (BBB) according to the yolk of the boiled egg; ^c^ Predicts the gastrointestinal absorption according to the white of the boiled egg; ^d^ Predicts the solubility of each compound in water; ^e^ Predicts the cytochrome P450 inhibition; ^f^ Predicts the bioavailability score.

**Table 4:** Predicted toxicity profile of 5-Tris and 6-Tris.

Compound	5-Tris	6-Tris
**Mutagenicity**	Mutagen	Mutagen
**Carcinogenicity (mouse)**	Positive	Positive
**Carcinogenicity (rat)**	Negative	Negative
**hERG inhibition (cardiotoxicity)**	Ambiguous	Moderate Risk

## Supplementary Materials

Supplementary Materials can be found at https://www.mdpi.com/2076-2607/8/2/293/s1.
