# Supplementary material for: Discovery of Two Brominated Oxindole Alkaloids as Staphylococcal DNA Gyrase and Pyruvate Kinase Inhibitors via Inverse Virtual Screening
Source: Microorganisms. 2020 Feb 20;8(2):293. doi: 10.3390/microorganisms8020293 (PMC7074965; doi:10.3390/microorganisms8020293)
Supplement: Supplementary file 1 [file microorganisms-08-00293-s001.pdf]

Supporting Information

Discovery of two brominated oxindole alkaloids as Staphylococcal DNA gyrase and pyruvate kinase inhibitors *via* inverse virtual screening.

Ahmed M. Sayed[1], Hani A. Alhadrami[2,3], Seham S. El-Hawary[4], Rabab Mohammed[5], Hossam M. Hassan[5], Mostafa E. Rateb[5,6], Usama R. Abdelmohsen[7,8], Walid Bakeer[9,*]

Table S1. Isolated marine-derived compounds (1 – 36) with their antibacterial activity in μM.

No	Structure	Name	Chemical Class	MIC (MRSA)	MIC (*B. subtilis*)	MIC (*E. coli*)	MIC (*P. aeruginosa*)
1	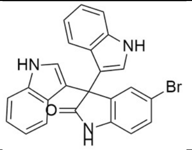	5-Bromotrisindoline	Indole	8	4	>256	>256
2	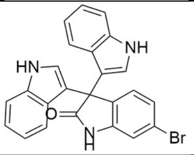	6-Bromotrisindoline	Indole	4	4	>256	>256
3	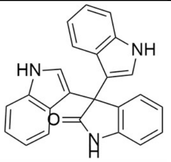	Trisindoline	Indole	32	16	>256	>256
4	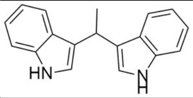	Vibrindole	Indole	64	64	128	>256
5	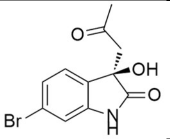	Convolutamidine F	Indole	128	128	>256	>256
6	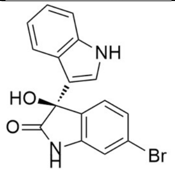	(S) 6-bromo-3-hydroxy-3-(1H-indol-3-yl) indolin-2-one	Indole	>256	128	>256	>256
7	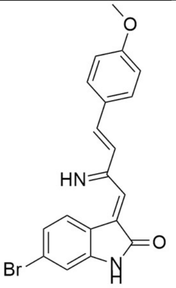	Saccharomonosporine A	Indole	>256	>256	>256	>256
8	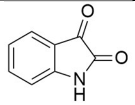	Isatine	Indole	256	>256	>256	>256

9	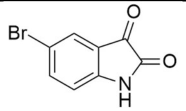	5-Bromoisatine	Indole	128	128	>256	>256
10	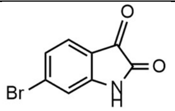	6-Bromoisatine	Indole	128	128	128	>256
11	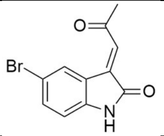	(E)-5-bromo-3-(2-oxopropylidene)indolin-2-one	Indole	>256	>256	>256	>256
12	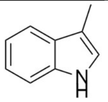	Skatole	Indole	>256	>256	>256	>256
13	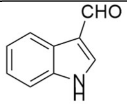	Indole-3-carbaldehyde	Indole	>256	>256	>256	>256
14	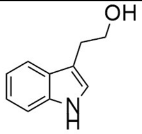	Tryptophol	Indole	>256	>256	>256	>256
15	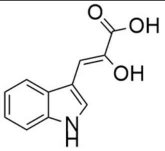	Indole-3-pyruvic acid	Indole	>256	>256	>256	>256
16	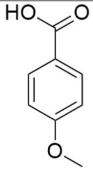	*p*-Anisic acid	Phenolic	>256	>256	>256	>256
17	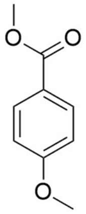	*p*-Anisic acid methyl ester	Phenolic	>256	>256	>256	>256
18	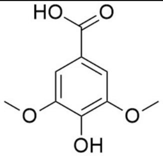	Syringic acid	Phenolic	>256	>256	>256	>256
19	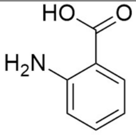	Anthranilic acid	Phenolic	>256	>256	>256	>256
20	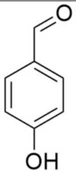	*p*-Hydroxy benzaldehyde	Phenolic	>256	>256	>256	>256

21	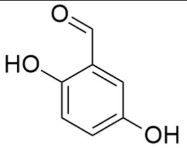	Gentisaldehyde	Phenolic	>256	>256	>256	>256
22	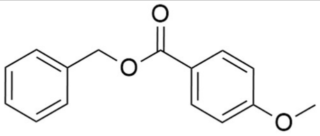	Benzyl anisate	Phenolic	>256	>256	>256	>256
23	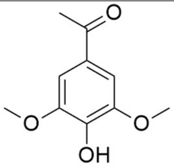	Acetosyringone	Phenolic	>256	>256	>256	>256
24	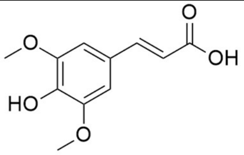	Sinapic acid	Phenolic	>256	>256	>256	>256
25	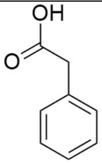	Phenyl acetic acid	Phenolic	>256	>256	>256	>256
26	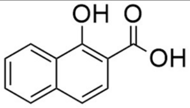	1-Hydroxy-2-naphthoic acid	Phenolic	>256	>256	>256	>256
27	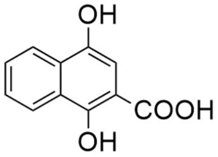	1,4 Dihydroxy-2-naphthoic acid	Phenolic	>256	>256	>256	>256
28	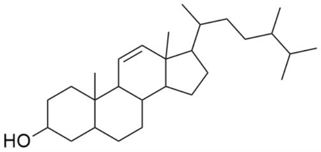	Callysterol	Steroid	>256	>256	>256	>256
29	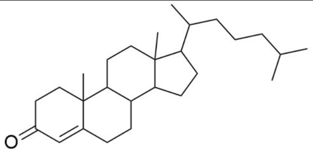	Cholestenone	Steroid	>256	>256	>256	>256
30	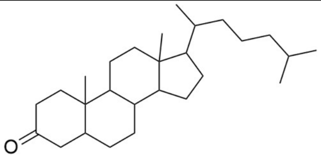	5α-Cholestanone	Steroid	>256	>256	>256	>256
31	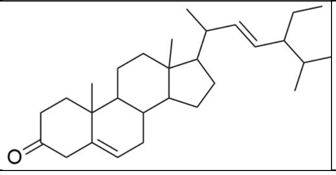	Stigmasterone	Steroid	>256	>256	>256	>256

32	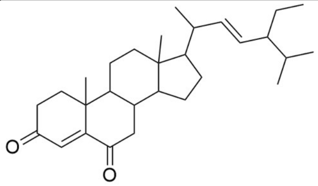	Stigmasta-4,22-dien-3,6-dione	Steroid	>256	>256	>256	>256
33	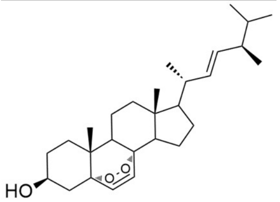	Ergosterol peroxide	Steroid	>256	>256	>256	>256
34	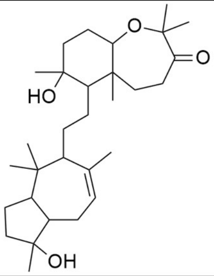	Sipholenone A	Triterpenoid	>256	>256	>256	>256
35	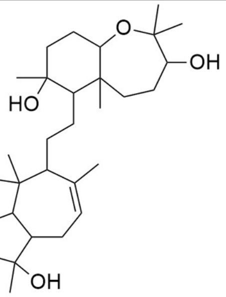	Sipholenol A	Triterpenoid	>256	>256	>256	>256
36	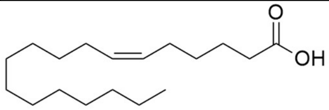	Petroselenic acid	Fatty acid	>256	>256	>256	>256

TableS2. Panel of antistaphylococcal targets used for Autodock-Vina calculations.

Target Name	PDB code	Resolution (Å)	Grid box (Å)
FtsZ	3VOB	2.7	center_x = 89.2672<br>center_y = 15.3214<br>center_z = 66.7351
PK	3T0T	3.1	center_x = 28.876<br>center_y = -1.32<br>center_z = -18.317
SpsB	4WVJ	1.95	center_x = 33.1403<br>center_y = -1.2257<br>center_z = 41.6906
Isoleucyl-tRNA synthetase	1JZS	2.5	center_x = -28.8564<br>center_y = 6.1865<br>center_z = -28.7767
Pdf	1Q1Y	1.9	center_x = -16.642<br>center_y = 145.0451<br>center_z = 47.0853
rRNA methyltransferase	4FAK	1.7	center_x = 13.4291<br>center_y = 23.5745<br>center_z = 25.993
Threonyl-tRNA synthetase	1NYQ	3.2	center_x = 53.7211<br>center_y = 42.4097<br>center_z = 78.337
Gyr A	2XCT	3.4	center_x = 40.965<br>center_y = 20.9118<br>center_z = 42.6244
Gyr B	3g7b	2.3	center_x = 48.559<br>center_y = -2.319<br>center_z = 20.417
ParE	4URN	2.3	center_x = 20.0552<br>center_y = 23.0763<br>center_z = 51.3535
Ddl	2I87	2	center_x = 16.3904<br>center_y = -3.8579<br>center_z = -0.7003
MurB	1HSK	2.3	center_x = 179.9707<br>center_y = 148.9228<br>center_z = 163.3638
PBP2	5M18	1.98	center_x = -13.5476<br>center_y = -20.9507<br>center_z = 62.345
DHFR	2W9H	1.48	center_x = 18.3749<br>center_y = 68.9681<br>center_z = 43.475
YycG/YycF	5IS1	2	center_x = 18.5417<br>center_y = 52.3793<br>center_z = 10.5854
FabF	2GQD	2.3	center_x = -23.9705<br>center_y = 32.8015<br>center_z = 4.5101
FabI	4CV1	1.95	center_x = 3.8691<br>center_y = -9.732

			center_z = 38.5248
LigA	4GLX	1.9	center_x = 2.4277<br>center_y = -19.8806<br>center_z = 56.7041
TrxB	4GCM	1.8	center_x = 26.6282<br>center_y = 30.8226<br>center_z = -12.0442
